# Evidence of secular variation in Archean crust formation in the Eastern Indian Shield

**DOI:** 10.1038/s41598-022-18372-9

**Published:** 2022-08-18

**Authors:** Prantik Mandal

**Affiliations:** CSIR-National Geophysical Research Institute, Hyderabad, Telangana 500076 India

**Keywords:** Solid Earth sciences, Seismology

## Abstract

Understanding the dominant crustal accretion model in any Archean craton is the key to understanding the dominant geodynamic process responsible for early crust formation during the Hadean (> 4.0 Ga) and Archaean (4.0–2.5 Ga). The continental crust has been proposed to have formed through either horizontal/vertical accretion related to subduction or mantle plume tectonic processes. Here, the Moho depths and average crustal Vp/Vs ratios are modelled at 16 broadband stations in the Eastern Indian Shield (EIS) through HK stacking of radial P-receiver functions (PRFs). These modelled parameters are used to test both plume and subduction models, which might have played a key role in the crustal accretion of the EIS throughout the Archean. We observe a correlation between crustal age and composition within the ellipsoidal Paleoarchean cratonic domain in the Singhbhum-Odisha-Craton (SOC), which reveals an increase in age from the younger granitoid core of the SOC (with thinning of felsic crust) to the surrounding older greenstone belts (with thickening of felsic crust). A thinner mafic crust resulting from multiple magmatic events characterizes the neighbouring Meso-Proterozoic Chotanagpur Granitic Gneissic terrain (CGGT). The Common Conversion Point (CCP) image of radial PRFs reveals northward subduction of the Paleoarchean SOC below the Meso-Proterozoic CGGT.

## Introduction

Seismic studies in different Archean cratons in the world have shown that a crustal thickness of 32–39 km with a low Vp/Vs ratio characterizes the old cratonic felsic crust (viz., mid-Archean or before Neo-Archean), which has been attributed to the delamination of dense, more mafic, and high-velocity lower crustal parts^1–3^. All Archean cratonic crusts are stabilized before 2.9 Ga^1^. However, a thicker and mafic (i.e. higher Vp/Vs ratio) crust characterizes the younger Archean cartons, suggesting a more mafic lower crustal composition^1,4,5^.

In the Kaapval craton, Africa, seismic studies have found a thin crust and sharp Moho, which has been attributed to the reworked crust resulting from lower crustal delamination caused by post-cratonization tectonothermal events^6–8^. The mid-Archean western Dharwar has been shown to be characterized by a thick crust (> 45 km) and diffusive Moho^9^. Similarly, Archean cratons in the European platform, Siberia and Greenland are also characterized by a thick crust (> 40 km)^10^.

Crustal and lithospheric structure in the Eastern Indian Shield (EIS), where the oldest Hadean rocks of 4.0–4.2 Ga^11,12^ were found, have been investigated using various seismic techniques, suggesting that vertical tectonics (plume activities) controlled the Neo-Archean crustal accretion at the Singhbhum-Odisha-Craton (SOC), as evidenced by modelled marked crustal and lithospheric thinning^13^. Geological evidence and modeling of P-receiver functions (PRFs) as well as S-receiver functions (SRFs) have shown northward subduction of the SOC under the Chotanagpur Granitic Gneissic Terrain (CGGT)^13–15^. Available Moho depths vary from 33 to 45 km in the SOC, 36–39 km in the Eastern Ghat Mobile Belts (EGMB), and 40–42 km in the CGGT, while the lithospheric thicknesses range from 98 to 140 km in the SOC, 114–122 km in the EGMB, and 128–140 km in the CGGT^13^. Using various geophysical techniques, a marked lateral variation in lithospheric thicknesses has also been detected across the EIS, e.g., 65 km by modelling heat flow data^16^, 70–100 km by modelling SRFs^17^, and 58–96 km by modelling magneto-telluric data^18^. Thus, the above-discussed estimates of lithospheric thicknesses suggest a much thinner lithosphere than typical cratons (> 250 km^19^), which has been attributed to the delamination of lithosphere below the SOC^14,18^.

Here, we investigate the crustal structure and composition through the HK stacking of radial PRFs at 16 broadband stations in the EIS. The region consists of two cratonic parts, the Archean SOC and Proterozoic CGGT, which enables us to study the crustal accretion processes during the period ranging from 4.2 to 1.0 Ga, resulting from both plume tectonics (vertical accretion) and subduction processes (horizontal accretion).

## Geological puzzles related to early crustal accretion

**Figure 1:**
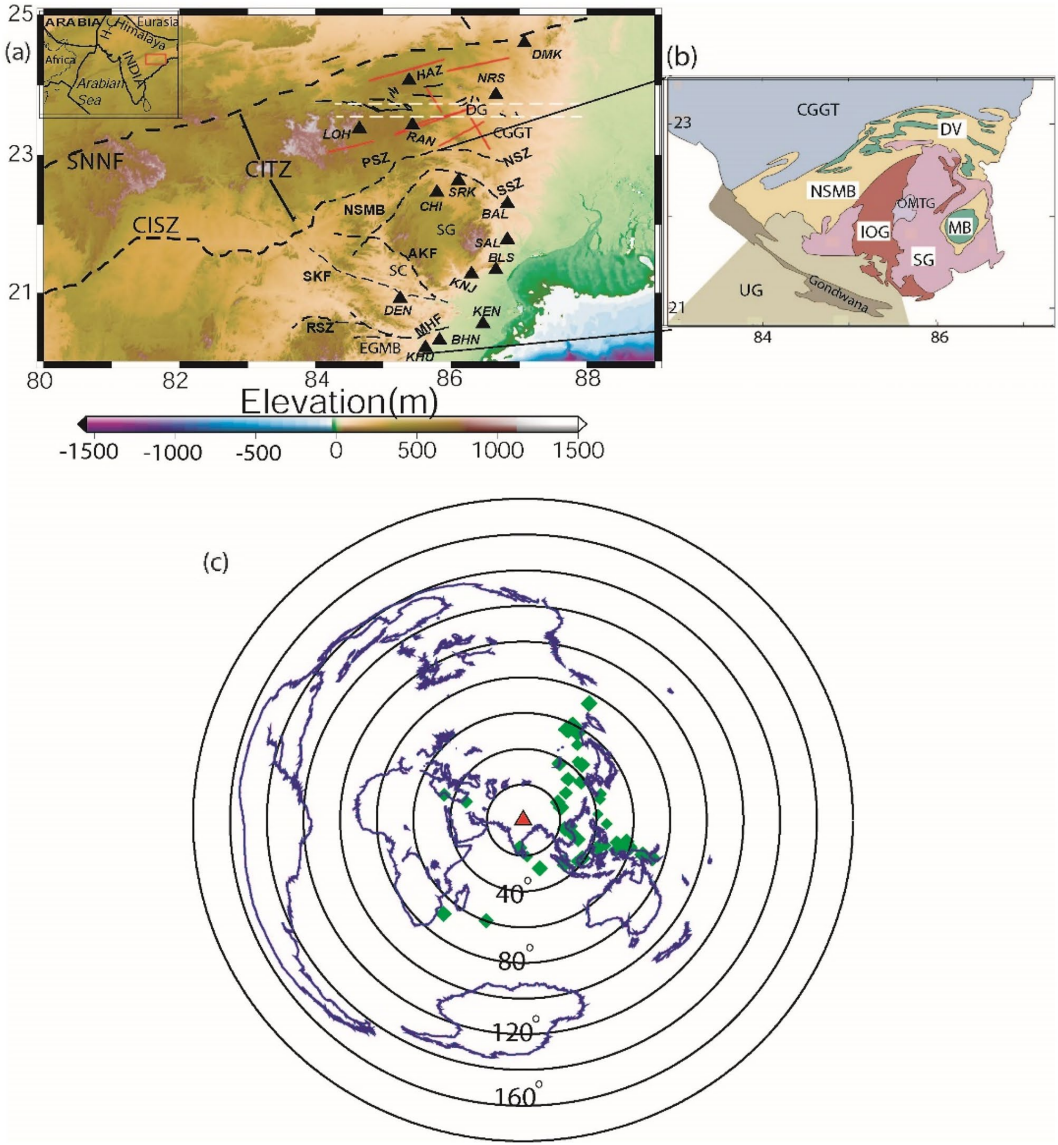
(**a**) Elevation (in m) map showing station distribution in the Eastern Indian shield. Filled black triangles mark the broadband seismograph stations (KHU-Khurda, BHN-Bhubaneswar, KEN-Kendrapara, DEN-Denkanal, KNJ-Keonjhor, BLS-Balasore, SAL-Salbani, BAL-Balukuria, CHI-Chaibasa, SRK-Saraikela, LOH-Lohardaga, RAN-Ranchi, HAZ-Hazaribagh, NRS-Nirsa, and DMK-Dumka). Black dotted lines mark major lithospheric fractures viz. CITZ (central Indian Tectonic zone), CISZ (central Indian shear zone) and SNNF (son Narmada north fault). SC, CGGT, SG, DG (white dotted lines) and EGMB represent the Singhbhum craton, Chotanagpur Granitic Gnessic terrain (eastern part of the CITZ), Singhbhum Granite (eastern part of the SC), Damodar graben and Eastern Ghats mobile belt, respectively. The dotted black line represents faults, the shear zone, and the lineament. AKF: Ankul fault; SKF: Sukhinda fault; RSZ: Ranipathar shear zone; MHF: Mahanadi fault; SSZ: south Singhbhum shear zone; NSZ: north Singhbhum shear zone; PSZ: Purulia shear zone. NSMB marks the north Singhbhum mobile belt. The solid gray lines represent lineaments, and the black lines represent faults in the Chotanagpur Plateau (upper part of the map). The inset shows the key map for the area, where the study area is shown by a red open square. H-C marks the Herat-Chaman plate boundary. (**b**) Geological map of the Singhbhum-Odisha Craton (SOC). OMG: Older metamorphic group; OMTG: Older metamorphic tonallite gneiss; SG; Singhbhum granite; DV: Dalma volcanic; MB: Metabasics; UG: Unclassified granite/granulite. (**c**) The epicentral plot of 150 teleseismic events, whose broadband data from our Singhbhum network are used for our receiver function study. A red triangle and green diamond symbols mark the center of our network (Lat. 85.6°, Long. 20.2°) and epicenters of selected teleseismic events.

The oldest zircon xenocrysts of 4.2 Ga in India have been found in the Paleoarchean TTGs of the SOC, which suggests that the oldest Archean (Hadean) crust was formed in this part of the Indian subcontinent^12^. Thus, the SOC is the only part of India where some imprints of the Hadean or Paleo-Neo Archaean crust could possibly exist, which were probably affected by both vertical and horizontal tectonic regimes. With the help of the geology and new U–Pb zircon age constraints on EIS, many authors have made efforts to study the crustal accretion processes responsible for forming the ellipsoidal Paleo-Meso-Archean granite greenstone nucleus and the flanking Proterozoic supracrustal cover sequences (Fig. 1a, b)^11,20–25^. In the north, the cratonic nucleus is bounded by the Phanerozoic Damodar Valley, while it is bounded by the Mahanadi Valley grabens in the southwest. Additionally, the Singhbhum cratonic nucleus is bounded by the Proterozoic EGMB in the southeast. The Singhbhum shear zone (SSZ) and north Singhbhum mobile belt (NSMB) characterize the northern limit of the cratonic nucleus, comprising ~ 3.52–3.29 Ga Older metamorphic tonalite gneiss (OMTG), Singhbhum granite (SG) and the Older metamorphic group (OMG)^20,26,27^ (Fig. 1a, b). The 3.51–3.33 Ga supracrustal rock successions (greenstones), the Iron Ore Group (IOG) occupies the surrounding region of the central granite-gneiss domain. The evidence of a magnetism episode at 3.47 Ga, 3.35 Ga and a last phase at 3.30 Ga have been observed in the cratonic nucleus^23^. The cratonization age of the SOC is found to be ~ 3.1 Ga. Based on geological and geochemical evidence, both plume-related^23^ and plate tectonic-style^28^ models have been proposed to explain the evolution of the SOC.

Several tectonic events have been discussed in the literature, which were responsible for shaping the EIS spanning from the Hadean to Neoproterozoic^13^. From 4.2 to 3.6 Ga, crust formation was mainly through the recycling of Hadean and Eoarchean crustal fragments in the SOC and was followed by the thickening of the oceanic mafic plateau due to plume episodes at 3.6 Ga. Then, at 3.52–3.38 Ga, the formation of OMTG, OMG, and IOG resulted from the melting of the mafic plateau. Subsequently, the Singhbhum granitoids were formed through magmatism episodes at 3.36–3.28 Ga. Stabilization of the craton took place at 3.18–3.09 Ga, while the sedimentation of Keonjhar took place at 2.9–2.8 Ga. Subsequently, the north Singhbhum shear zone was formed at 2.45–2.0 Ga, while the Dhalbhum, Dalma and Chandil Formations formed as accretionary orogens at 1.8–1.6 Ga. Following this event, northern and southern Indian cratons were separated along the Central Indian Tectonic Zone (CITZ) at 1.6–1.3 Ga. Finally, the advancing subduction zone and re-amalgamation of the southern Indian cratons with the northern Indian cratons along the CITZ took place at 1.3 to 1.0 Ga^13^.

## Seismic network, earthquake data and modelling

During 2013–2016, a local-cum-regional seismic network of 16 broadband seismic stations (Fig. 1a; Table 1) with an average interstation spacing of 40 ± 15 km was operated by the National Geophysical Research Institute under the Council of Scientific and Industrial Research, India. The network data^13^ enabled us to perform the H–K stacking^29^ of radial P-receiver functions (PRFs) for estimating crustal thicknesses and average crustal Vp/Vs ratios in the EIS. Each station was equipped with a 24-bit Reftek-130 recorder and a 120 s Reftek broadband sensor. A GPS receiver clock at each station was used for time tagging, while a recording speed of 50 samples per second was used to record continuous broadband data.

**Table 1:** Modelled Moho depths and crustal composition in the eastern Indian craton, through the HK stacking of PRFs and lithospheric thicknesses through the joint inversion of PRFs and group velocity surface wave dispersion data^13^.

Station	Latitude (^o^N)	Longitude (^o^E)	Vp/Vs	Poisson’s ratio	Moho Depth (km)	Lithospheric thickness (km) (Mandal et al., 2021)	Nature of the crust
DEN	20.92	85.25	1.69	0.23	45.1	135	Felsic
KNJ	21.28	86.30	1.69	0.23	45.0	135	Felsic
BLS	21.34	86.66	1.70	0.24	44.0	135	Felsic
SAL	21.78	86.83	1.68	0.23	45.1	140	Felsic
CHI	22.46	85.79	1.95	0.32	33.6	98	Mafic
BAL	22.30	86.83	1.88	0.30	35.6	136	Mafic
SRK	22.63	86.11	1.66	0.22	45.6	116	Felsic
KHU	20.20	85.62	1.69	0.23	45.3	122	Felsic
KEN	20.55	86.47	1.84	0.29	41.7	122	Mafic
BHN	20.31	85.83	1.95	0.32	33.6	114	Mafic
RAN	23.43	85.43	1.67	0.23	46.5	135	Felsic
HAZ	24.07	85.38	1.65	0.21	46.7	134	Felsic
NRS	23.86	86.66	1.85	0.29	35.9	140	Mafic
DMK	24.60	87.08	1.97	0.33	32.4	128	Mafic
LOH	23.37	84.65	2.07	0.35	29.5	140	Mafic
RAM	23.76	86.83	2.11	0.36	28.4	–	Mafic

Here, three-component broadband waveforms of 150 good teleseismic events of m_b_ ≥ 5.5 (with back azimuth between 38° and 300°, epicenters between 30°S and 90°N, and ray parameters ranging from 0.040 to 0.080 s/km) from 16 stations are used for the PRF study (Fig. 1c). First, a from − 5 to 60 s is considered from the 3-component broadband data, which is corrected from the instrumental correction. Then, all the windowed waveforms are filtered using a high pass filter with a corner frequency of 0.02 Hz before the computation of PRFs. Here, the iterative time domain deconvolution procedure of Ligorria and Ammon^30^ with 200 iterations is used to compute radial and transverse PRFs for each event, using a Gaussian width factor, a = 2.5 (f < 1.25 Hz), where “f” marks the corner frequency of a low-pass filter. Thus, the wavelegth would be 4.4 km for f = 1.25 and an average crustal shear velocity of 3.5 km/s. Thus, the calculated PRFs with a = 2.5 can resolve layers with thicknesses of 2.2 km. Finally, we selected deconvolutions that reproduced more than 90% of the signal energy on the radial component (when convolved back with the vertical trace).

A total of 666 individual radial PRFs were computed using 2000 three-component waveforms from all 16 broadband stations (see Supplementary Figs. S1, S2). In this study, a minimum of 15 and a maximum of 60 individual radial PRFs at 16 different stations are used to estimate the Moho depths through HK stacking. The stacked individual radial P-RFs at 16 broadband stations (see Supplementary Figs. S2a-p, S3-11) reveal that there are at least three prominent phases corresponding to conversions from the Moho (P_ms_) and crustal multiples (P_p_P_ms_ and P_s_P_ms_ + P_p_S_ms_ phases). The Moho conversion (P_ms_) and first crustal multiple (P_p_P_ms_) show a positive conversion representing a velocity increase across the Moho, while the second crustal multiple (P_s_P_ms_ + P_p_S_ms_) shows a negative conversion representing a decrease across the Moho (see Supplementary Figs. S2–S11). The minimum arrival time of P_ms_ is observed at CHI station, while the maximum arrival time of P_ms_ is observed at SAL station (see Supplementary Figs. S2–S11). The back-azimuthal variations of PRF images at different stations along with theoretical arrivals of s-to-p conversion from Moho and crustal multiples are shown in Supplementary Figs. S7–S11. Radial PRF gather are also shown at different stations, showing arrivals of the Moho conversion and crustal multiples (Figs. S7-S11). Finally, the arrival times and amplitudes of the P_ms_, P_p_P_ms_ and P_s_P_ms_ + P_p_S_ms_ phases from individual radial PRFs estimated for different back azimuths at sixteen broadband sites are used as inputs for the HK stacking of radial PRFs. In our analysis, we set w_1_ = 0.7, w_2_ = 0.2 and w_3_ = 0.1 for some stations, while most of the stations gave better results for w_1_ = 0.34, w_2_ = 0.33, and w_3_ = 0.33. For our study, we vary the H values from 20 to 70 km and the k values from 1.5 to 2.2. Finally, those measurements of (H, k) showing a clear closure between H and k are selected. Additionally, we used different velocities varying from 6.3 to 6.5 km/s for stacking.

**Figure 2:**
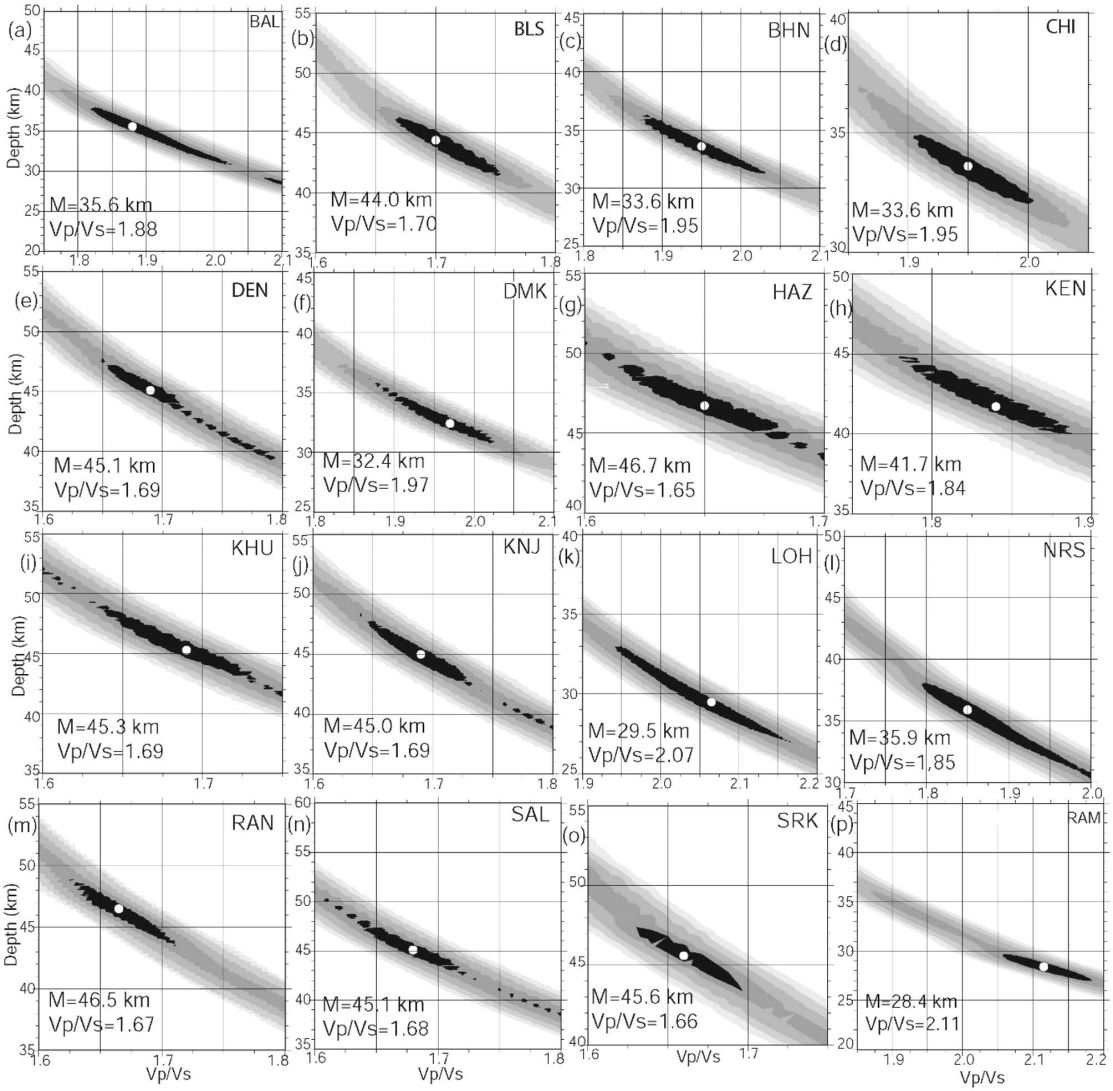
H–K Stacking of PRFs at 16 broadband seismograph sites in the EIS: **(a**) BAL, (**b**) BLS, (**c**) BHN, (**d**) CHI, (**e**) DEN, (**f**) DMK, (**g**) HAZ, (**h**) KEN, (**i**) KHU, (**j**) KNJ, (**k**) LOH, (**l**) NRS, (**m**) RAN, (**n**) SAL, (**o**) SRK and (**p**) RAM. The best estimated H and K values are indicated by a small white filled circle at the centre of the black error ellipse.

**Figure 3:**
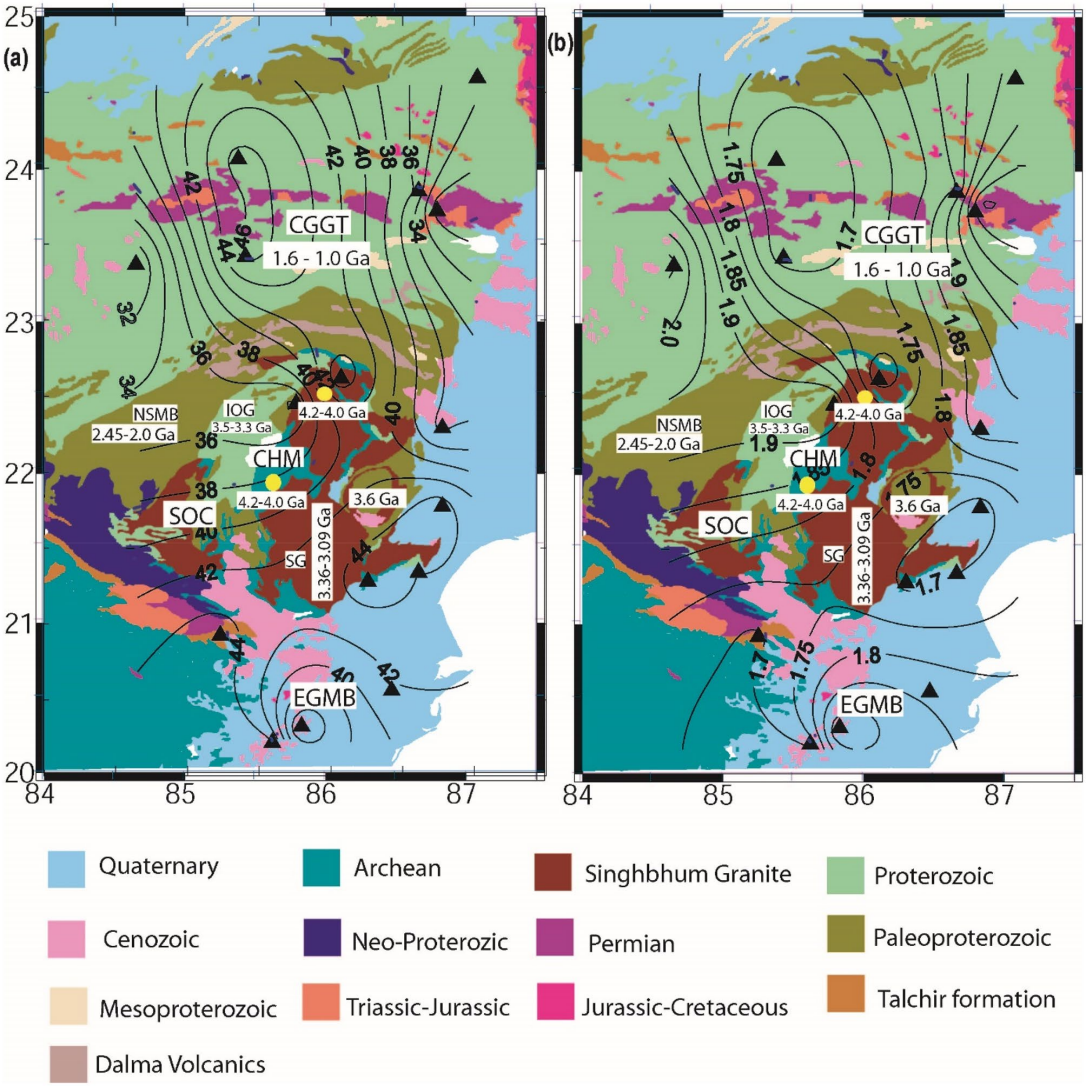
Contour plots of modelled (**a**) Moho depths (in km) and (**b**) average crustal Vp/Vs ratios. Black filled triangles mark 16 broadband stations in the EIS. Different geological formations of the EIS are shown in different colours. SOC, CGGT, and EGMB mark the Singhbhum-Odisha Craton, Chotanagpur Granitic Gneissic terrain, and Eastern Ghats Mobile belt, respectively. IOG and SG represent the Iron Ore Group supracrustals and Singhbum Granite, respectively. CHM shows the location of Champua. Two yellow filled circles (A and B) mark the locations of samples of 4.2–4.0 Ga TTGs^11,12^.

**Figure 4:**
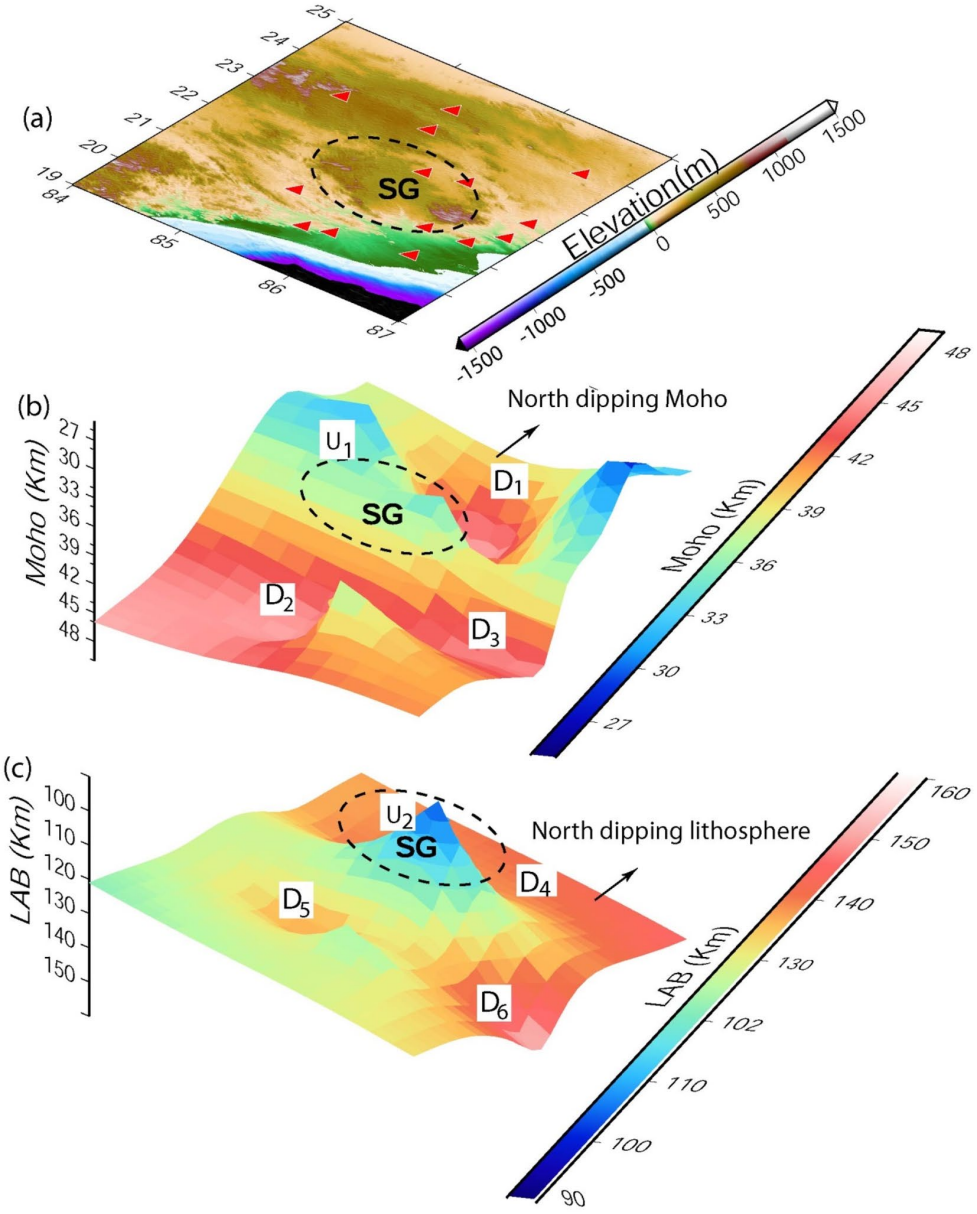
(**a**) Elevation (in m) map along with seismic stations (marked by red filled triangles). 3-D surface plots of modelled (**b**) Moho (km) and (**c**) lithospheric thickness (in km). The central part of the Singhbhum Odisha Craton (SOC) is marked by black dotted elliptical area below which maximum crustal and lithospheric thinning is modelled while arrow marks the North dipping Moho and lithosphere. U1 and U2 mark the zones of upwarping of the Moho and lithosphere, respectively, below the central granitoid of the SOC. Below the peripheral zones of the SOC, the zones of crustal thickening are marked by D1, D2 and D3 and the zones of lithospheric thickening are represented by D4, D5 and D6.

The H and k values are estimated through the HK stacking of radial PRFs at 16 broadband stations within the measuring errors (± 0.45 km and 0.025 for H and k, respectively; Fig. 2a–p). The contours of our estimated Moho depths (in km) and average crustal Vp/Vs values are shown in Fig. 3a, b. A 3-D structural model for the EIS based on our modeling results is also proposed in Fig. 4a–c. Modelled Moho depths, Vp/Vs and Poisson ratios at 16 broadband stations are listed in Table 1. The lithospheric thicknesses estimated by Mandal et al.^13^ are also listed in Table 1.

### Common conversion point (CCP) stacking of PRFs

**Figure 5:**
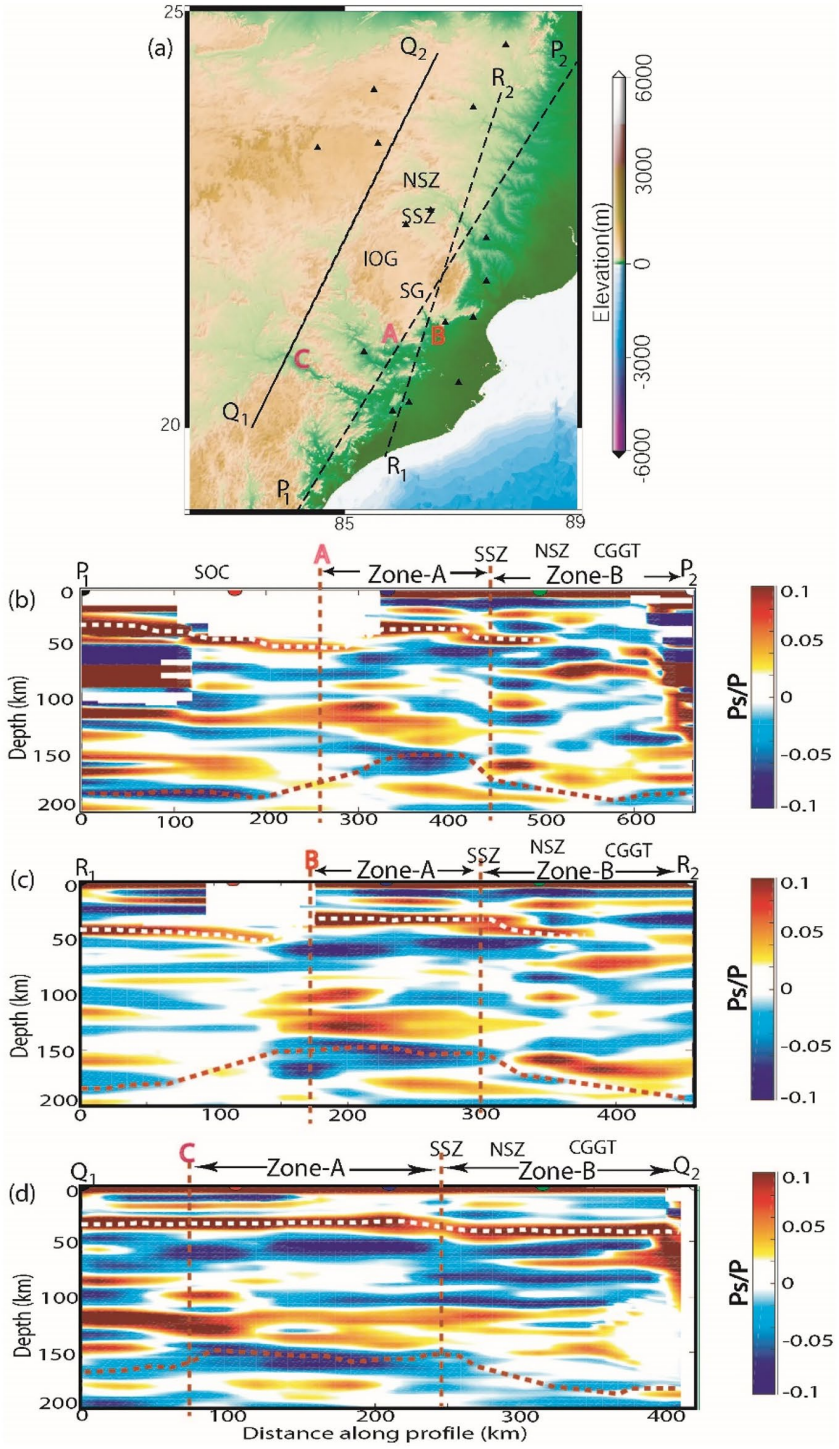
Common Conversion Point (CCP) stacking of radial PRFs (**a**) along three NE-SW trending profiles (P_1_P_2_, Q_1_Q_2_ and R_1_R_2_), (**b**–**d**) CCP images along P_1_P_2_, Q_1_Q_2_ and R_1_R_2_ profiles, respectively, showing a north dipping subduction of Singhbhum craton below the Chotanagpur Granitic Gnessic terrain (CGGT). Yellow dotted line marks the Moho depth variation while red dotted line shows the variation of lithosphere-asthenosphere boundary across the Eastern Indian Shield (EIS). The zone-A between the point A, B, and C and the south Singhbhum Shear zone (SSZ) on the profiles P_1_P_2_, Q_1_Q_2_ and R_1_R_2_, respectively, shows the region with marked crustal and lithospheric thinning due to 3.5 Ga and the 1.6 Ga plume activities that spatially correlates well with the Singhbhum granite (SG) and Iron Ore Group (IOG) basin. And, the zone-B between the SSZ and the region north of it (i.e. CGGT) is characterized by the crustal and lithospheric thickening indicating the zone of subduction of the SOC below the CGGT. NSZ marks the north Singhbhum shear zone.

Here, we use the Funclab software^31^ to perform CCP imaging of radial PRFs, using Dueker and Sheehan^32^’s methodology to coherently stack p-to-s phase conversions for generating a 2-D image of impedance contrast at depth. The details of CCP imaging methodology have been discussed in Cladwell et al.^33^ and the manual of Funclab^31^. Here, we used the 1-D IASP91 velocity model for the CCP imaging^34^. We have performed CCP imaging along five profiles (two along and three across the Himalayan collisional zone), whose locations are shown in Fig. 1a. The results of CCP stacking of radial PRFs along seven NE-SW and N-S trending profiles are shown in Fig. 5a–d and Supplementary Figs. S12a–d. These CCP images show lateral variations in the modelled Moho and LAB below the Eastern Indian Shield.

We compute PRFs using three frequency bands corresponding to Gaussian width factors, a = 1.0 (f < 0.5 Hz), and a = 2.5 (f < 1.25 Hz). Thus, the wavelegth would be 8, 4 km for f = 0.5 and 1.0, respectively, for an average crustal shear velocity of 4.0 km/s. Thus, the calculated PRFs with a = 1.0 and 2.5 can resolve layers with thickness of 4 and 1.75 km, respecively. The nominal vertical resolution at the Moho could be 4 km for the 1-D PRFs and 1.7 km for 2-D CCP stack^33^. While the horizontal resolution depends on the frensel zone. The fresnel zone width for Ps at 40 km is ~ 40 km. The station spacing for our stations varies from 10 to 20 km. So our station spacing of ~ 20 km could yield 50% overlap at 40 km depth, which is found to be suffiicient to image the Moho well^33^. At 10 km depth, the frensel zone width is 20 km, which could be image with 50% overlap for our stations with a 10 km spacing. But, our station spacing could not provide beller resolution for images at depths less than 10 km.

Following above-discussed CCP imaging technique, we generate crustal images by stacking radial PRFs. Our CCP image (Fig. 5a–d and see Supplementary Figs. S12a–d) shows a marked crustal as well as lithospheric thinning below the region covering the central Singhbhum granitoid (SG) and the north Singhbhum mobile belt (NSMB) and then both the Moho and Lithosphere deepen down toward the north below CGGT. Our modelling detects a crust-mantle boundary at 40–45 km depths beneath the region south of the SOC and thins to 35–40 km depth below the region between SG and NSMB and finally deepens to 42–48 km depths below the CGGT (Fig. 5a–d). Our modelling also detects a lithosphere-asthenosphere boundary (LAB) conversion at 160–180 km depth below the region south of the SOC (where we do not have any station) and then it thins to a depth of 130–150 km below the region between SG and NSMB and finally it deepens to 170–200 km depth below the CGGT, suggesting a northward subduction of the SOC below the CGGT (Fig. 5a–d; see Supplementary Figs. S12a–d). Note that our earlier studies using the PRF modelling^35^ and joint inversion^14^ of the PRFs and surface wave group velocity dispersion data have also shown a northward subduction of the SOC below the CGGT.

## Results and conclusions

Lateral variations in Moho depths and average crustal compositions have been modeled across the EIS through the H–K stacking of radial P-receiver functions at 16 individual broadband locations (Table 1). The estimated Moho depths and average crustal Vp/Vs ratios vary from 33.6 km and 1.95 (at CHI and BHN) to 45.6 km and 1.66 (at SRK) in the SOC, while these estimates in the CGGT vary from 28.4 km and 2.11 (at RAM) to 46.7 km and 1.65 (at HAZ). The thinnest crust of 33.6 km (with Vp/Vs of 1.95) in SOC is obtained at the CHI and BHN stations (Table 1). CHI is located in the region influenced by the 1.6 Ga Dalma volcanism, while BHN lies in the EGMB, which was influenced by the ~ 117 Ma Rajmahal volcanism. While the thickest crust of 45.6 km (with Vp/Vs of 1.66) is modeled at SRK, which is located in the region near the northern periphery of the SOC, our modeling reveals a thicker felsic crust (41.7–45.6 km) in the older peripheral regions of the Singhbum carton (viz., SRK, BLS, DEN, KHU, KNJ, SAL and KEN stations) and a thinner and mafic crust of ~ 32 km (with Vp/Vs of 2.0) in the region west of the Champua (CHM), which is spatially correlated with the exposed Dalma volcanics (Fig. 3). Figure 4b, c show marked crustal and lithospheric thinning underlying the central granitoid of the SOC, which supports the model of vertical accretion above the Archean plume, affecting the upper mantle zone^13,20^. The zones of crustal and lithospheric thickening characterize the peripheral greenstone belts of the SOC (Fig. 4b, c). We observe that the mean Moho depths, lithospheric thickness, Vp/Vs value and Poisson’s ratio are found to be 42 ± 5 km, 128 ± 5 km, 1.75 ± 0.12, and 0.25 ± 0.04 in the SOC, respectively, while they are found to be 40 ± 6 km, 119 ± 5, 1.83 ± 0.13, and 0.28 ± 0.05 in the EGMB, respectively (Table 1). In the EGMB, modeled crustal thicknesses and crustal Vp/Vs ratios vary from 33.6 km and 1.95 (at BHN) to 45.3 km and 1.69 (at KHU), respectively. These results get further support from the findings of our earlier studies using PRFs in the EIS^14,35^ suggesting an average crustal velocity of (4.0 ± 0.08), (4.03 ± 0.06) and (3.9 ± 0.01) below the SOC, EGMB and CGGT, respectively, which, in turn, suggests a more mafic crust in the SOC and EGMB, than the relatively less mafic crust below the CGGT.

In CGGT, the thinnest crust of 28.4 km thickness is modeled at RAM, which is located in the Gondwana Basin coalfields (Figs. 2, 3). The thickest crust at 46.7 km is modeled at HAZ, which is located near the northern end of the CGGT. In the central part of the Proterozoic, the CGGT-occupying HAZ and RAN stations show thicker felsic crust (Vp/Vs ~ 1.6–1.7). The modelled thinning of mafic crust (Vp/Vs ~ 1.8–2.0) at the LOH station in the western part of the CGGT could be due to the 1.6 Ga mantle plume activity associated with Dalma volcanism (Fig. 3a, b). From Fig. 3, another two regions of mafic crustal thinning are noticed below the EGMB and the region northeast of the study area in the CGGT, which could be attributed to the Rajmahal volcanism of ~ 117 Ma.

The modeled crustal Vp/Vs ratios (Fig. 3b) reveal a marked lateral variation in the crustal composition across the EIS. The modelled average crustal Vp/Vs and Poisson’s ratio estimates vary from 1.6 to 2.08 and 0.18 to 0.35, respectively. Christensen^36^ suggested that minerals that exhibit Vp/Vs ratios higher than 1.8 include plagioclase, amphibole, pyroxene, and Fe-olivine. Furthermore, Christensen^36^ has shown that Fe substitution for Mg in pyroxene and olivine also increases the Vp/Vs ratio; thus, basic compositions are expected to result in higher Vp/Vs ratios. Note that felsic rocks with intermediate to high silica contents could result in low Vp (< 6.7 km/s) and low Vp/Vs (< 1.78), while anorthosite rocks with high plagioclase contents have been shown to have relatively low Vp (between 6.6 and 7.1 km/s) and high Vp/Vs (> 1.85). However, mafic rocks with low silica contents could yield high Vp (> 6.7 km/s) and high Vp/Vs ratios (up to 1.86). Higher Vp/Vs values exceeding 1.86 could be possible in the presence of high-pressure fluids/melts in subduction zones^37,38^. The high Poisson’s ratio values of 0.32 at CHI and 0.35 at LOH along with crustal and lithospheric thinning suggest the presence of crustal metamorphic fluids below the region west of the study area, spatially correlating with the region with exposed Dalma volcanics^25^. The larger Poisson’s ratio of 0.29 at KEN and 0.32 at BHN in the EGMB along with crustal and lithospheric thinning suggests the presence of crustal metamorphic fluids and densification of the lower crust due to magmatic episodes associated with Rajmahal volcanism. The surrounding regions (occupying DEN, KNJ, BLS, BAL, SAL, and SRK) of the Singhbhum Cratonic nuclei are modelled to be characterized by felsic crust (0.18–0.24). In the eastern CGGT, larger Poisson’s ratios of 0.35 at NRS and 0.33 at DMK along with thin crust and thicker lithosphere depict the presence of dense lower crust and metamorphic fluids related to the magmatic activity associated with the Proterozoic subduction episode. The smaller Poisson’s ratios of 0.23 at RAN and 0.21 at HAZ in the central CGGT suggest a relatively thick felsic crust.

Our modeling reveals remarkable seismic signatures within the ellipsoidal Paleoarchean cratonic domain. The central granitoid core region comprising a relatively younger SG is modelled to be characterized by a thinner felsic crust, while the peripheral regions are characterized by a thicker felsic crust, except the western peripheral side, which is characterized by the thinning of a mafic crust (Vp/Vs ~ 1.8–2.0) (Fig. 3a, b). The suggested melting of the oceanic mafic plateau model to create OMTG and IOG in the SOC at 3.52–3.38 Ga^23,24,28^ is further supported by our results showing uniformly thin and felsic crust throughout the SOC region, which may dominate the initial crust formation in the Paleoarchean SOC. The primary signatures of earlier magmatism/volcanism episodes have been reported to be preserved as eruptive rocks and meta-sediments in the IOG and Simlipal basin^28^. Multiple magmatic pulses in a confined region have been suggested by the existing geochoronological data, where successive magmas signifying the granitoid batholiths follow the same conduit, resulting in pushing the older plutonic elements to the periphery of the ellipsoidal cratonic domain (involving Rayleigh–Taylor instabilities and gravitational overturns of the crust^13,39^) to form the “keel and basin” structure of the granite-and-greenstone terranes at the surface^13^. This process has been best documented in the case of the Pilbara craton, Australia^38^. Further, the multiple magmatic pulses might have successively melted the crust and subsequently generated more felsic crust in the SOC (Fig. 3a, b). Furthermore, based on the low Cu in the Paleoproterozoic diamictites, it has been proposed that the upper continental crust became dominated by felsic rocks at the end of Archean^40,41^. Additionally, the preferential weathering of exposed mafic components might have led to the felsic Archean upper continental crust in the SOC containing lavas and intrusions, basalts, and ultramagnesian (Mgo > 18%) komatiites of 3.25 Ga^42,43^.

The neighbouring Meso-Proterozoic CGGT does not show any evidence of a “keel and basin” structure but rather provides support for the terrane accretion model that apparently worked during 1.6–0.9 Ga^15^. The mean Moho depth, lithospheric thickness, Vp/Vs value and Poisson’s ratio are found to be 37 ± 8 km, 135 ± 5 km, 1.89 ± 0.21, and 0.30 ± 0.06 in the CGGT, suggesting that a thinner mafic crust characterizes the CGGT (Table 1). The geochronological data have shown that the crustal block underlying the CGGT has been influenced by at least four magmatic-metamorphic events at > 2.5, 1.6–1.5, 1.2–1.0, and 0.9 Ga^44,45^. These events might have resulted in modeled mafic and thin crust in the CGGT. Furthermore, the presence of tholeiitic trends for basalt and calc-alkaline affinities of andesite, rhyolite and granite in the Gaya district of CGGT supports the idea of their generation in an island arc, subduction-related setting during the Neoproterozoic (1.0 Ga)^46^. Our 3-D structural plot (Fig. 4a–c) also depicts a north-dipping Moho as well as lithosphere beneath the EIS, suggesting northward subduction of the SOC below the CGGT. Furthermore, our CCP images along three NE-SW trending profiles (i.e. P1P2, Q1Q2 and R1R2 as shown in Fig. 5a–d) across the EIS detect a distinct zone-A (as shown in Fig. 5b–d) of crustal as well as lithospheric up-warping below the region between the SG and SSZ on the P1P2, Q1Q2 and R1R2 profiles, respectively, and this thinning model could be attributed to the 3.5 Ga^20^ and 1.6 Ga^24^ mantle plume episodes. This thinning model gets further support from the presence of ultramagnesian (Mgo > 18%) komatiites of Archean age (~ 3.25 Ga) in the Badampahar-Gorumahisani Greenstone Belt in the SOC^43^. These komatiites were crystallized from the hot magma, which were generated by moderate-high degree melting of mantle plume at different depths during the Archean^43^. Figures 5(b-d) also detect a zone-B between the SSZ and region north of it (i.e. CGGT), which is characterized by marked crustal and lithospheric thickening, supporting the idea of northward subduction of the Archean SOC below the Meso-Proterozoic CGGT that is in good agreement with the earlier proposed Proterozoic accretion model of the EIS during 1.6–0.9 Ga^15^. The CCP images along two N-S (A_1_A_2_ and B_1_B_2_) and one NW–SE (C_1_C_2_) profiles (see supplementary Figs. S12a–d) also reveal similar images as seen in Fig. 5a–d suggesting a clear northward subduction of the SOC below the CGGT. Thus, our observations suggest that the imprints of the Paleoarchean crust formed above the mantle plume and its modification in the Proterozoic subduction process are still preserved in the EIS, which may be related to the secular cooling of the Earth’s mantle.

## Methods

### Estimation and H–K stacking of P-radial receiver functions

The amplitude and time of P-to-S conversions (Ps) from the Moho and reverberations associated with interfaces below a recording site are generally modelled by the receiver function method (Fig. S1). The direct P-waves are generally impulsive on the vertical component seismogram at epicentral distances exceeding 30°_,_ while Ps conversions dominate the horizontal components of ground motion (Fig. S1). The amplitude of conversion (Ps) and multiples (PpPms, PpSms, etc.) depends on the incidence angle of impinging P-waves and the size of the velocity contrasts at the interfaces. The P-wave incidence angle and ray parameter control the arrival times of conversions and multiples on the radial RF. All possible P-to-S conversions and multiples beneath a seismic station are shown in Fig. 2a–p, S1, S2a–p, and S3-S11.

Here, we study 666 good teleseismic earthquakes from 16 broadband stations (Fig. 1a, c). In this study, a minimum of 21 and a maximum of 35 individual radial RFs at 16 different stations are used to estimate the Moho depths through H–K stacking^29^ of radial PRFs. Before stacking, a moveout correction is applied using the modified IASP91 global reference model^38^ and a reference slowness of 6.4 s/° permitting summation of records from different distances. For the H–K stacking, we selected normal move-out corrected individual radial RFs (for different ranges of azimuths and epicentral distances for different stations). It is quite clear that the individual radial RFs show clear and sharp P-to-S conversions (Pms at 3.6–7.0 s after Pp (i.e., direct P arrival)) and some weak multiples (i.e., PpPms and PpSms + PsPms) from the crust–mantle boundary, suggesting that there is probably a clear Moho underlying the study region (Fig. 2a–p, see Supplementary Figs. S2a–p, S3–S11). We note that prominent Pms arrivals characterise all individual radial RFs from all 16 broadband stations (Fig. 2a–p, see Supplementary Figs. S2a–p, S3–S11), while some weak Moho multiples are also noticed in all individual radial RFs. Finally, the crustal Vp/Vs and Moho depths were estimated at 16 broadband stations through the H–K stacking^29^ of moveout corrected radial P-RFs over the available range of horizontal slowness and back azimuths (Table 1).

The moveout times are a function of the P-wave slowness (i.e., the distance), crustal thickness, and Vp/Vs ratio. They only weakly depend on the absolute average crustal P-wave velocity^16^. In this method, the arrival times of the P_ms_, P_p_P_ms_ and P_s_P_ms_ + P_p_S_ms_ phases are predicted using Eqs. (2–4), and weighting factors w_1_, w_2_, and w_3_ in Eq. (1) are chosen to balance the contributions from different phases, as mentioned above. Using radial PRFs from events at different distances and stacking all the appropriately shifted traces gives a robust estimate for the thickness of the crust and (with larger uncertainty) for the Vp/Vs ratio.

After obtaining the move-out corrected radial receiver functions for different stations (using Eqs. 2–4), the average crustal Vp/Vs and Moho depth (z_M_) were estimated using the approach of Zhu and Kanamori^29^. In this technique, a grid search over the Vp/Vs- z_M_ space is performed with a view to obtain the (Vp/Vs, z_M_) pair, which is the closest agreement with the observed P_ms_, P_p_P_ms_ and P_s_P_ms_ + P_p_S_ms_ phases. It is observed that these phases are quite clear on the estimated radial PRFs for almost all the stations (see Supplementary Figs. S1a-b, S2a–p).

Zhu and Kanamori^29^ outlined a simple method to estimate the crustal thickness and the average crustal Vp/Vs ratio. They used a migration scheme for the direct Ps conversions and the crustal multiples for a set of receiver functions, assuming crustal homogeneity. They defined a quantity S(H,k) representing the weighted sum of the receiver function amplitudes at the calculated times of predicted arrivals of Ps, PpPms and PpSms + PsPms, which is expected to be maximum for a correct combination of H and k. This quantity can be written as:

1 $${\text{S}}\left( {{\text{H}},{\text{k}}} \right){ } = \left[ {{\text{ w}}_{1} {\text{r}}_{{\text{j}}} \left( {{\text{t}}_{1} } \right) + {\text{w}}_{2} {\text{r}}_{{\text{j}}} \left( {{\text{t}}_{2} } \right){-}{\text{w}}_{3} {\text{r}}_{{\text{j}}} \left( {{\text{t}}_{3} } \right)} \right]$$

where w_1_, w_2_, and w_3_ are weighting factors, which are chosen to balance the contribution from three phases viz., Ps, PpPms and PpSms + PsPms. r_j_(t) is the amplitude of the receiver function for the j^th^ component, while t_1_, t_2_, and t_3_ are the predicted arrival times of the Ps, PpPms and PpSms + PsPms phases.

The moveout times for the respective phases are given by the following formulas:

2 $${\text{t}}_{{{\text{Ps}}}} = {\text{h(a}} - {\text{b)}}$$

3 $${\text{t}}_{{{\text{Pp}} + {\text{Ps}}}} = {\text{h(a}} + {\text{b)}}$$

4 $${\text{t}}_{{{\text{Pp}} + {\text{Ss}}}} = {\text{h2a}}$$

where h denotes the crustal thickness, and a and b are defined as:

5 $${\text{a}} = \left( {{\text{1/Vs}}^{{2}} {-}{\text{p}}^{{2}} } \right)^{{{1}/{2}}}$$

6 $${\text{b}} = \left( {{\text{1/Vp}}^{{2}} {-}{\text{p}}^{{2}} } \right)^{{1/2}}$$

and p is the horizontal ray parameter.

Furthermore, we model Poisson’s ratio from the estimated Vp/Vs values using the following relation:

7 $$\upsigma = \frac{[1-2\left(\frac{Vs}{Vp}\right)^2]}{2[1-\left(\frac{Vs}{Vp}\right)^2]}$$

## Supplementary Information

Supplementary Information.

## Data Availability

Most of the data that support the findings of this study are available in the supplementary material.
