# Supplementary material for: Evidence of secular variation in Archean crust formation in the Eastern Indian Shield
Source: Sci Rep. 2022 Aug 18;12:14040. doi: 10.1038/s41598-022-18372-9 (PMC9388659; doi:10.1038/s41598-022-18372-9)
Supplement: Supplementary file 1 — Supplementary Information. [file 41598_2022_18372_MOESM1_ESM.pdf]

Supplemental Information

Manuscript title:

Evidence of secular variation in Archean crust formation in the Eastern Indian Shield

Author: Prantik Mandal

This material includes a description of data and algorithms. It also presents the results of the HK stacking of radial PRFs. It includes 12 figures showing basic theory of the PRF, stacked radial PRFs, stacked radial as well as transverse PRFs at different stations and CCP imaging of radial PRFs along one NW-SE and two N-S profiles in the Eastern Indian Shield (EIS).

Data and Seismic Network

In 2013-2016, a seismic network consisting of 16 three-component broadband seismographs was installed by CSIR-NGRI, Hyderabad, in the Eastern Indian Shield (Fig.1a). Each station was equipped with a 24-bit Reftek-130 recorder, 120s Reftek 3-component broadband sensor and a GPS clock for time tagging. The data was recorded at 100 samples/s. The seismographs were located on hard rock sites for achieving higher signal to noise ratio. During February 2013 - February 2017, 2000 good teleseismic earthquakes were recorded by the 16 seismographs from the above network. For the present study, we used the above-mentioned dataset to compute radial PRFs through time domain deconvolution method of Ligorria and Ammon[1].

H-K stacking of radial PRFs

Here, we use 1500 radial P-receiver functions showing clear P-to-s conversions associated with the Moho and other crustal multiples (Fig. S1), which are estimated through the time domain deconvolution (with a Gaussian width=2.5) procedure of Ligorria and Ammon[2] with 200 iterations, from 16 broadband stations in the EIS (Fig. 1a), to conduct the H-K stacking of P-RFs (Figs. 2a-m). Stacked radial and transverse PRFs at 12 out of 16 stations are shown in Figs. S3-6, showing smaller amplitudes on stacked transverse PRFs, suggesting good radial PRFs. Further, the PRF imaging with back-azimuths and gathered

radial PRF at 13 stations are shown in Figs. S7-S11, which are also showing arrivals of theoretical converted phases from the Moho and crustal multiples. Due to the uneven azimuthal distribution of teleseismic events (Fig. 1b), reliable anisotropic structures are difficult to retrieve with this dataset, and so we invert only for isotropic crustal structure. For HK stacking of P-receiver functions[3], we used radial PRFs from 16 stations to delineate Moho depths and average crustal Vp/Vs values in the EIS region. The CCP images (generated using FUNCLAB software[4-5]) along two N-S and NW-SE trending profiles (supplementary Figs. S12a-d) suggest a northward subduction of the SOC below the CGGT.

References:

1. Mandal, P., Kumar, P., Sreenivas, B., Babu, E.V.S.S.K., Bhaskar Rao, Y.J. Variations in crustal and lithospheric structure across the Eastern Indian Shield from passive seismic source imaging: Implications to changes in the tectonic regimes and crustal accretion through Precambrian. Precambrian Research 360, 106207 (2021).
2. Ligorria, J.P., Ammon, C.J., 1999. Iterative deconvolution and receiver-function estimation. Bull. Seismol. Soc. Am. 89(5), 1395–1400.
3. Zhu, L. and Kanamori, H. (2000) Moho Depth Variation in Southern Caornia from Teleseismic Receiver Functions. Journal of Geophysical Research, 105, 2969-2980. http://dx.doi.org/10.1029/1999JB900322
4. K.C. Eagar, FuncLab: A MATLAB interactive toolbox for handling receiver function datasets. *Seis. Res. Lett.* 83, doi:10.1785/gssrl.83.3.596 (2012).
5. K.G. Dueker, A.F. Sheehan, Mantle discontinuity structure from midpoint stacks of converted P to S waves across the Yellowstone hotspot track. *J.Geophys. Res.* 102 (B4), 8313–8327, URL http://dx.doi.org/10.1029/96JB03857 (1997).

Supplementary information of 12 figures:

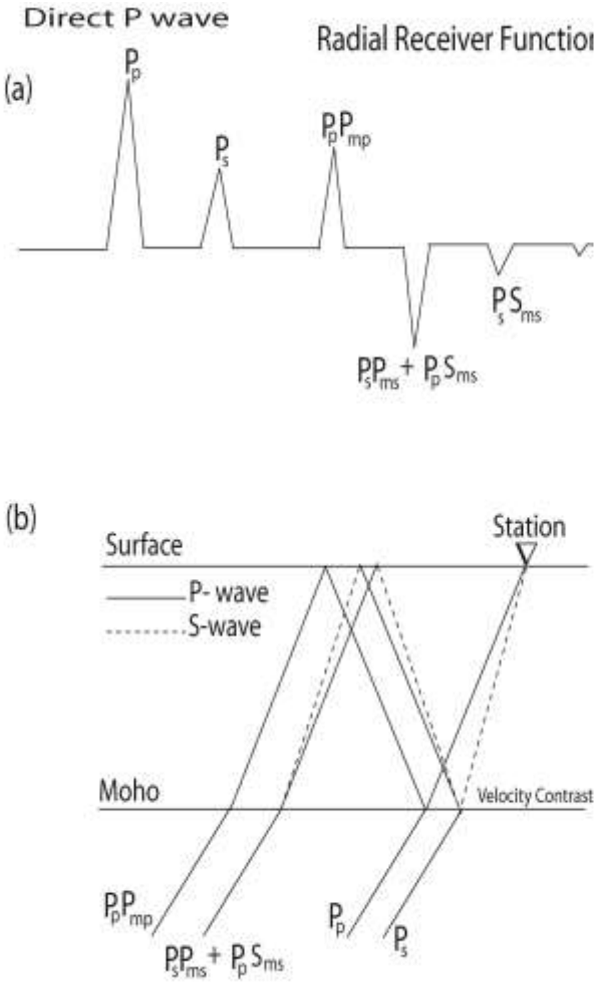

Figure S1: (a) A radial P-receiver function showing arrivals of various direct and converted phases of teleseismic P phases and (b) A plot showing raypaths of P-to-s conversions from the Moho and other crustal multiples.

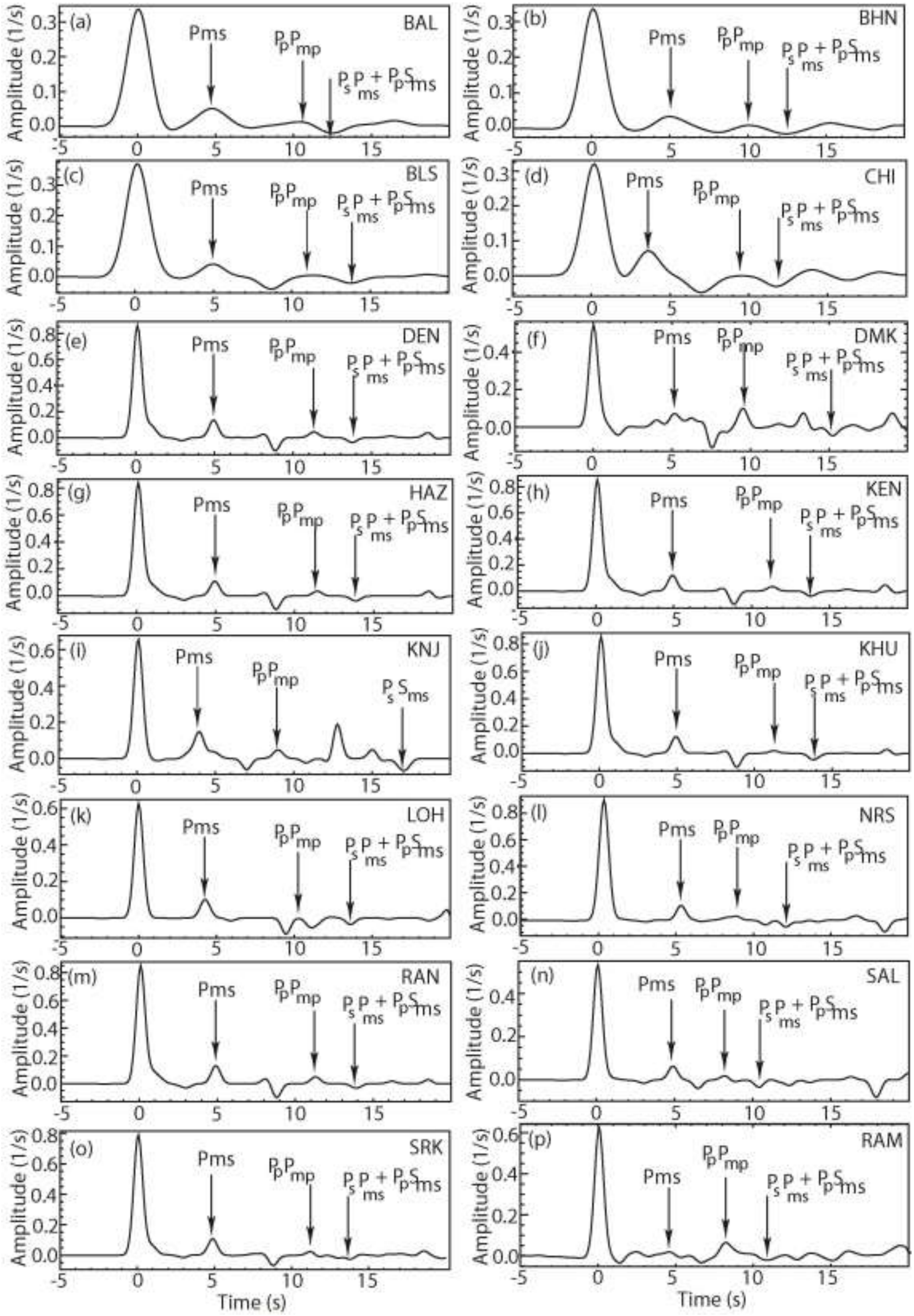

Figure S2: Stacked PRF showing conversions from the Moho (P_{ms}) and crustal multiples (P_pP_{mp}, (P_sP_{ms}+P_pS_{ms}), P_sS_{ms}) for 16 different stations in the EIS (a) BAL, (b) BHN, (c) BLS, (d) CHI, (e) DEN, (f) DMK, (g) HAZ, (h) KEN, (i) KNJ, (j) KHU, (k) LOH, (l) NRS, (m) RAN, (n) SAL, (o) SRK and (p) RAM.

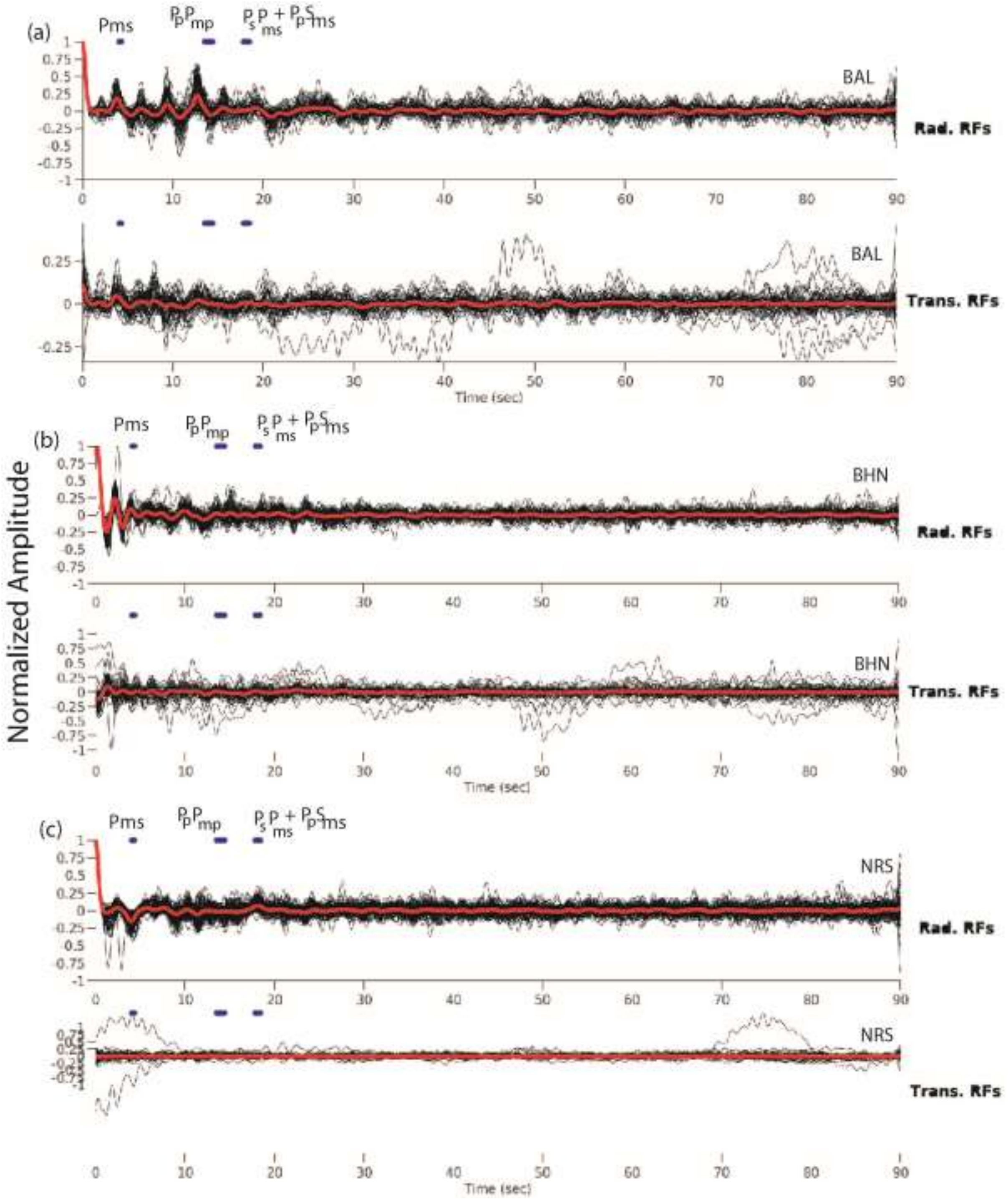

Figure S3: Stacked radial and transverse P-receiver functions at (a) BAL, (b) BHN and (c) NRS stations. Radial stacked PRF are showing conversions from the Moho (P_{ms}) and crustal multiples (P_pP_{mp}, (P_sP_{ms}+P_pS_{ms}), P_sS_{ms}). Stacked PRFs are shown by thick red lines while individual PRFs are shown by black thin lines. Theoretical arrivals of the Moho conversion and other multiples are marked by filled small black rectangles just above the stacked PRFs.

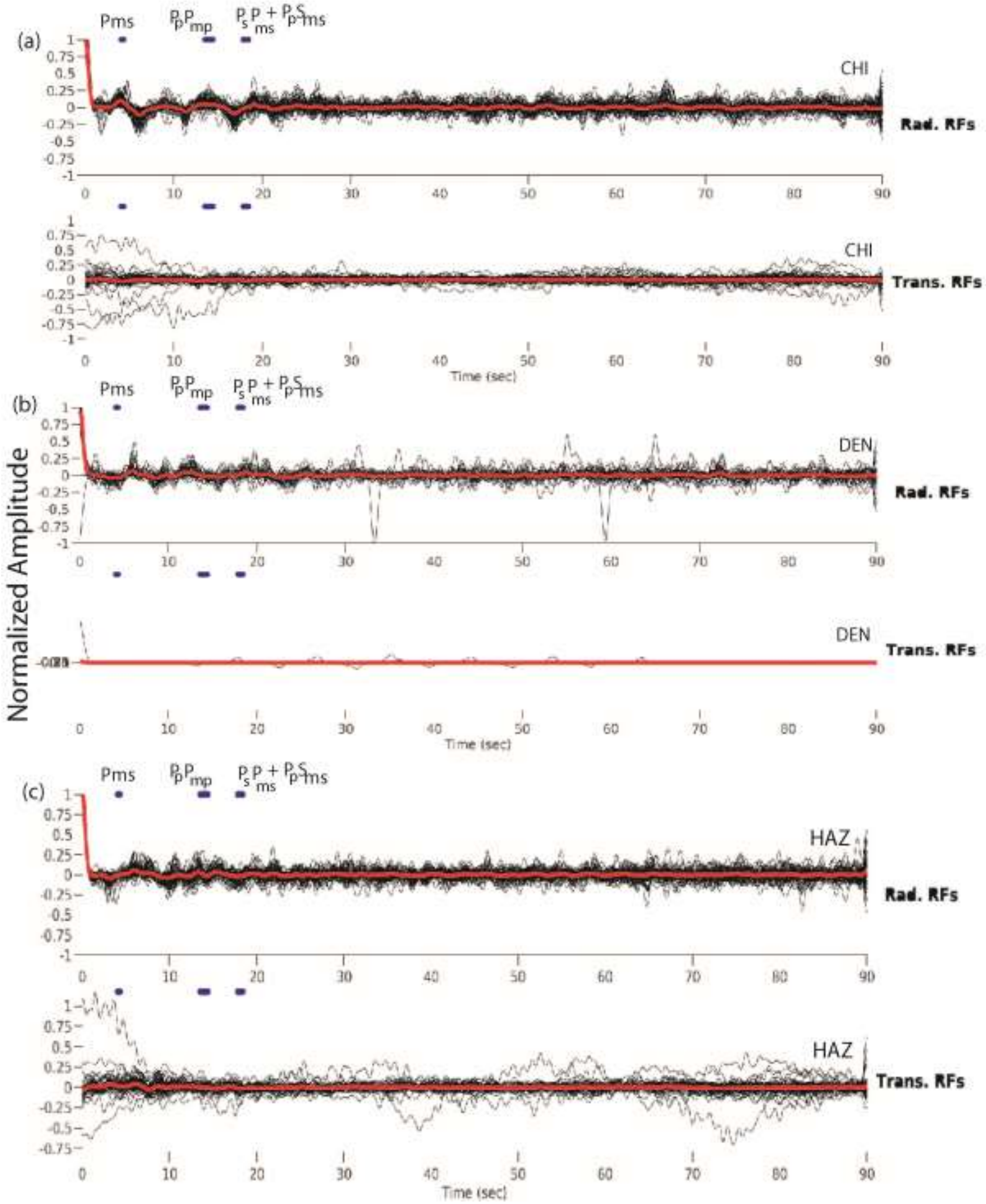

Figure S4: Stacked radial and transverse P-receiver functions at (a) CHI, (b) DEN and (c) HAZ stations. Radial stacked PRF are showing conversions from the Moho (P_{ms}) and crustal multiples (P_pP_{mp}, (P_sP_{ms}+P_pS_{ms}), P_sS_{ms}). Stacked PRFs are shown by thick red lines while individual PRFs are shown by black thin lines. Theoretical arrivals of the Moho conversion and other multiples are marked by filled small black rectangles just above the stacked PRFs.

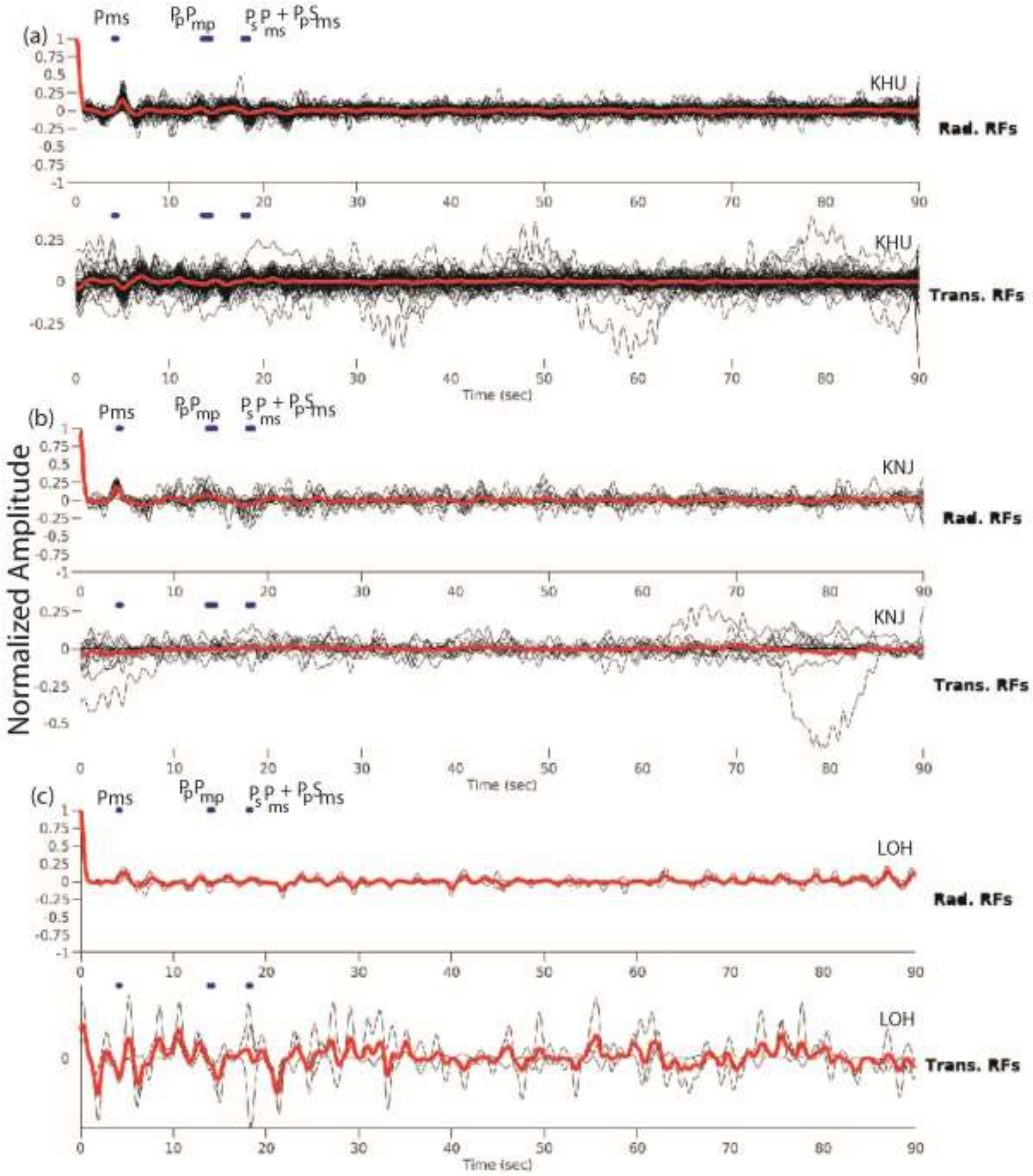

Figure S5: Stacked radial and transverse P-receiver functions at (a) KHU, (b) KNJ and (c) LOH stations. Radial stacked PRF are showing conversions from the Moho (P_{ms}) and crustal multiples (P_pP_{mp}, (P_sP_{ms}+P_pS_{ms}), P_sS_{ms}). Stacked PRFs are shown by thick red lines while individual PRFs are shown by black thin lines. Theoretical arrivals of the Moho conversion and other multiples are marked by filled small black rectangles just above the stacked PRFs.

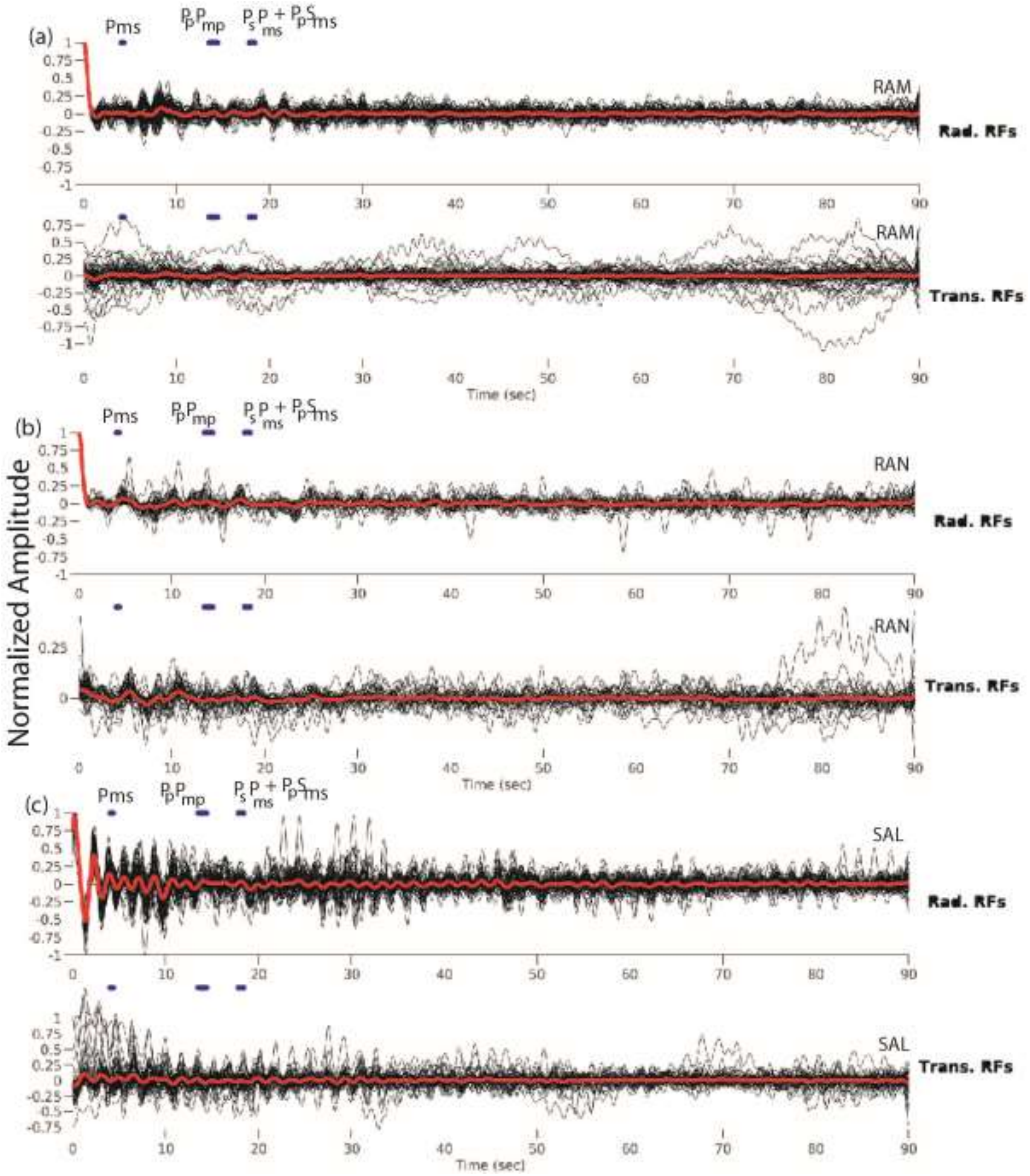

Figure S6: Stacked radial and transverse P-receiver functions at RAM, RAN and SAL stations. Radial stacked PRF are showing conversions from the Moho (P_{ms}) and crustal multiples (P_pP_{mp}, (P_sP_{ms}+P_pS_{ms}), P_sS_{ms}). Stacked PRFs are shown by thick red lines while individual PRFs are shown by black thin lines. Theoretical arrivals of the Moho conversion and other multiples are marked by filled small black rectangles just above the stacked PRFs.

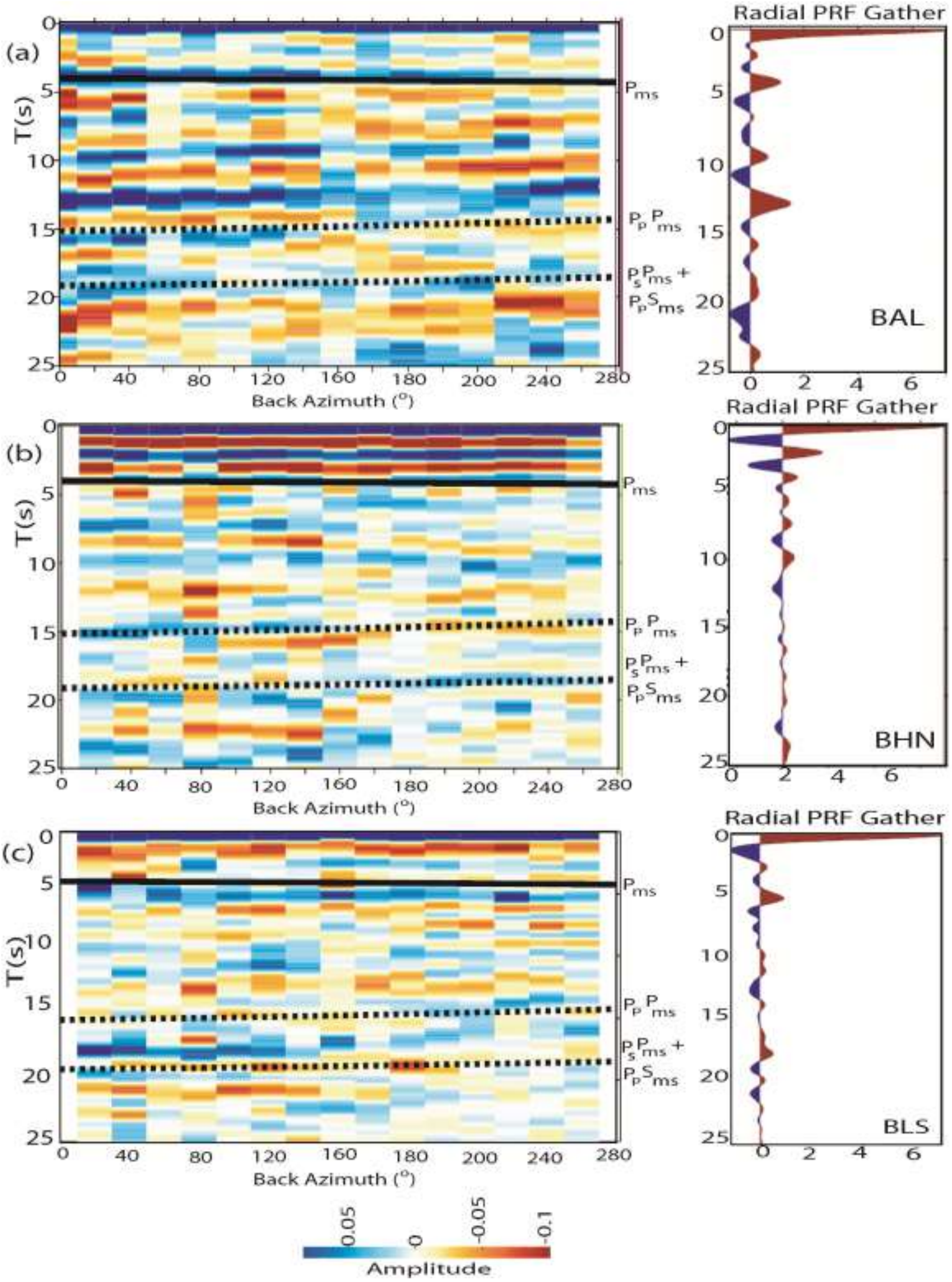

Figure S7: (a) The left panel of Fig. S7a shows the PRF Imaging with back-azimuth at BAL station showing arrivals of conversions from the Moho (Ps) and crustal multiples (PpPs and Ps+Ss). The theoretical times (using IASP91) of Ps conversion from the Moho are shown by black solid line while the same from crustal multiples (PpPs and Ps+Ss) are marked by black dotted lines. The right panel of Fig. S7a shows the radial PRF gather at BAL, (b) similar to Fig. S7a for the BHN station, (c) similar to Fig. S7a for the BLS station.

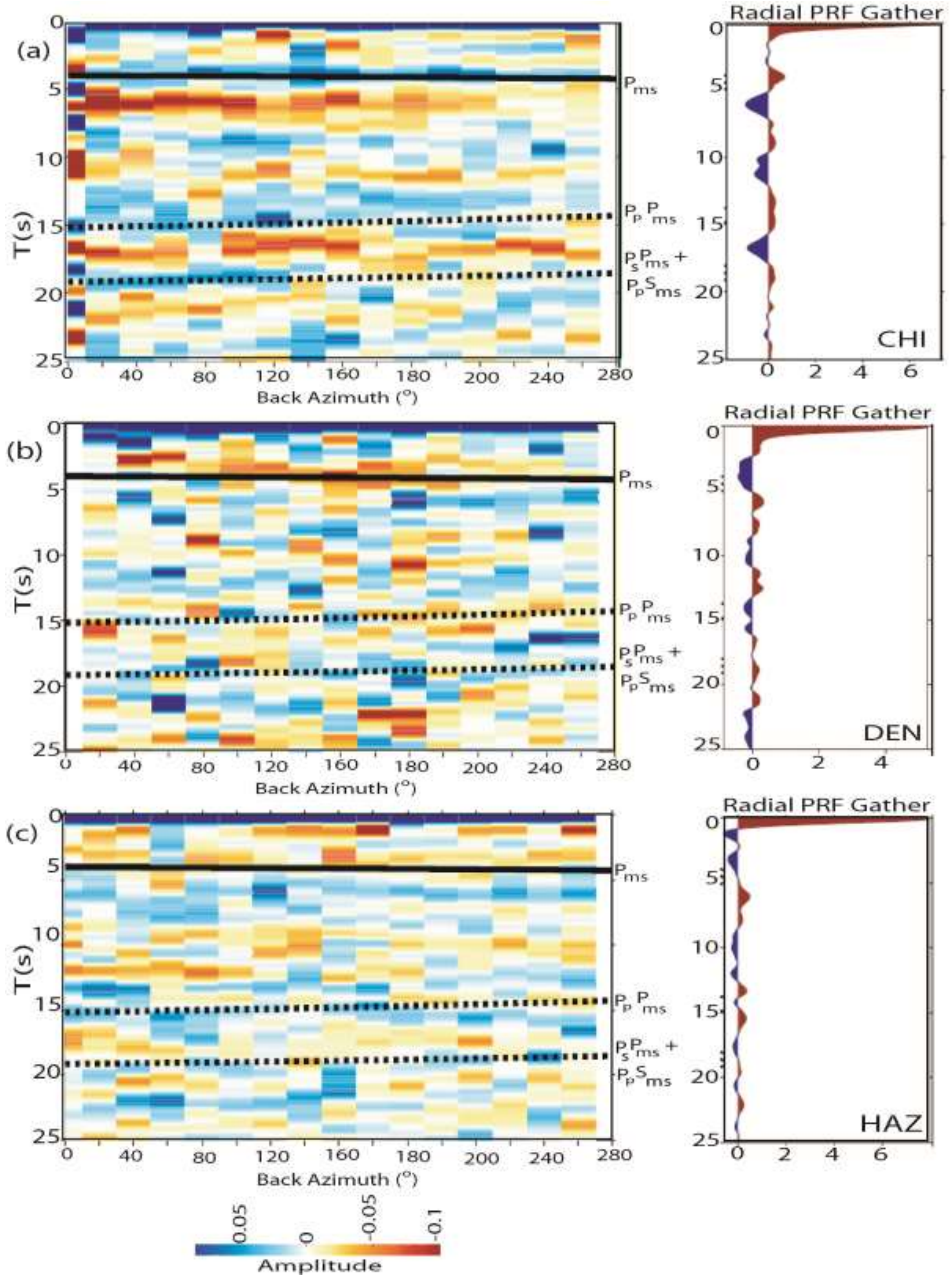

Figure S8: (a) similar to Fig. S7a for the CHI station, (b) similar to Fig. S7a for the DEN station, (c) similar to Fig. S7a for the HAZ station.

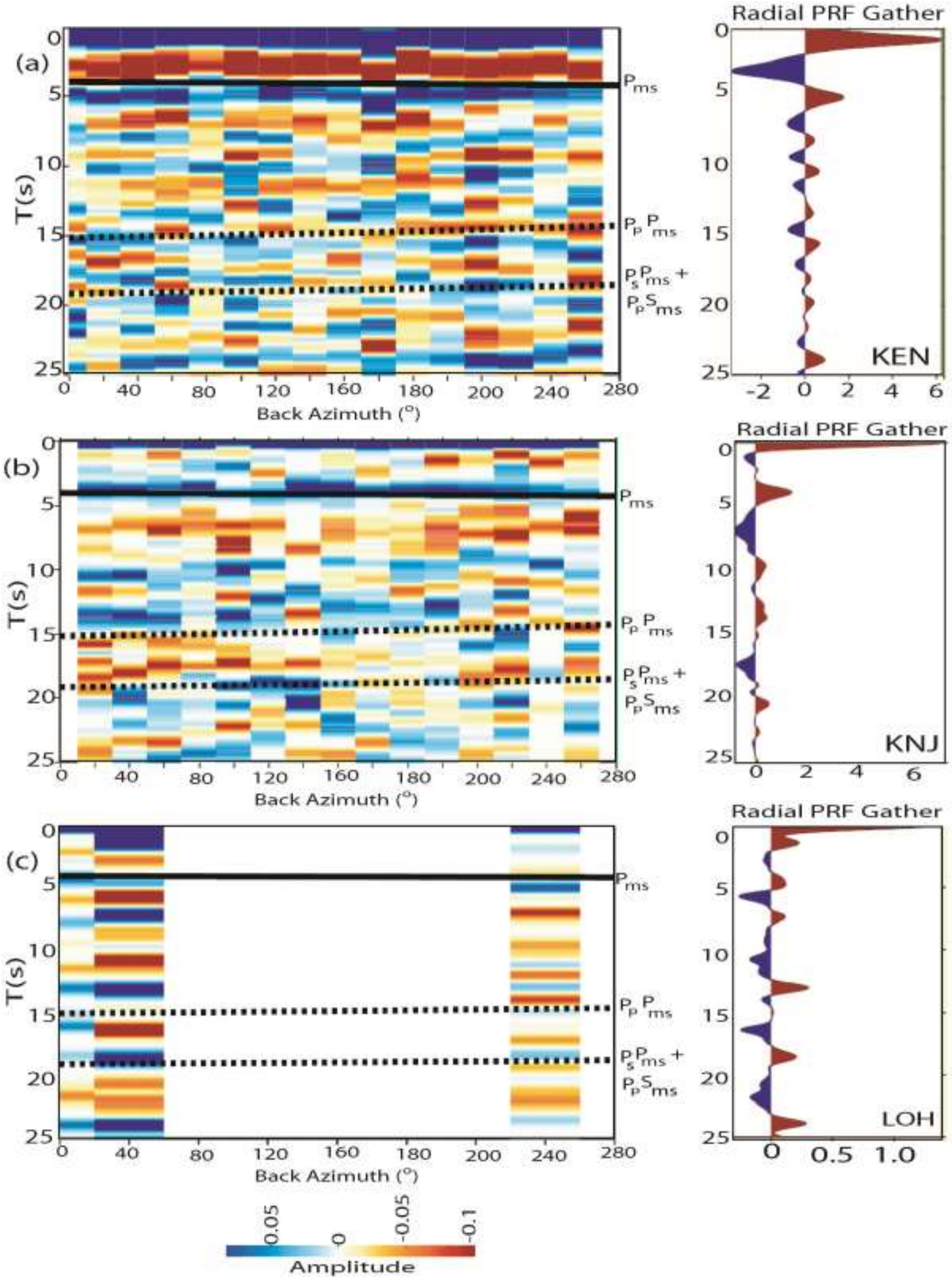

Figure S9: (a) similar to Fig. S7a for the KEN station, (b) similar to Fig. S7a for the KNJ station, (c) similar to Fig. S7a for the LOH station.

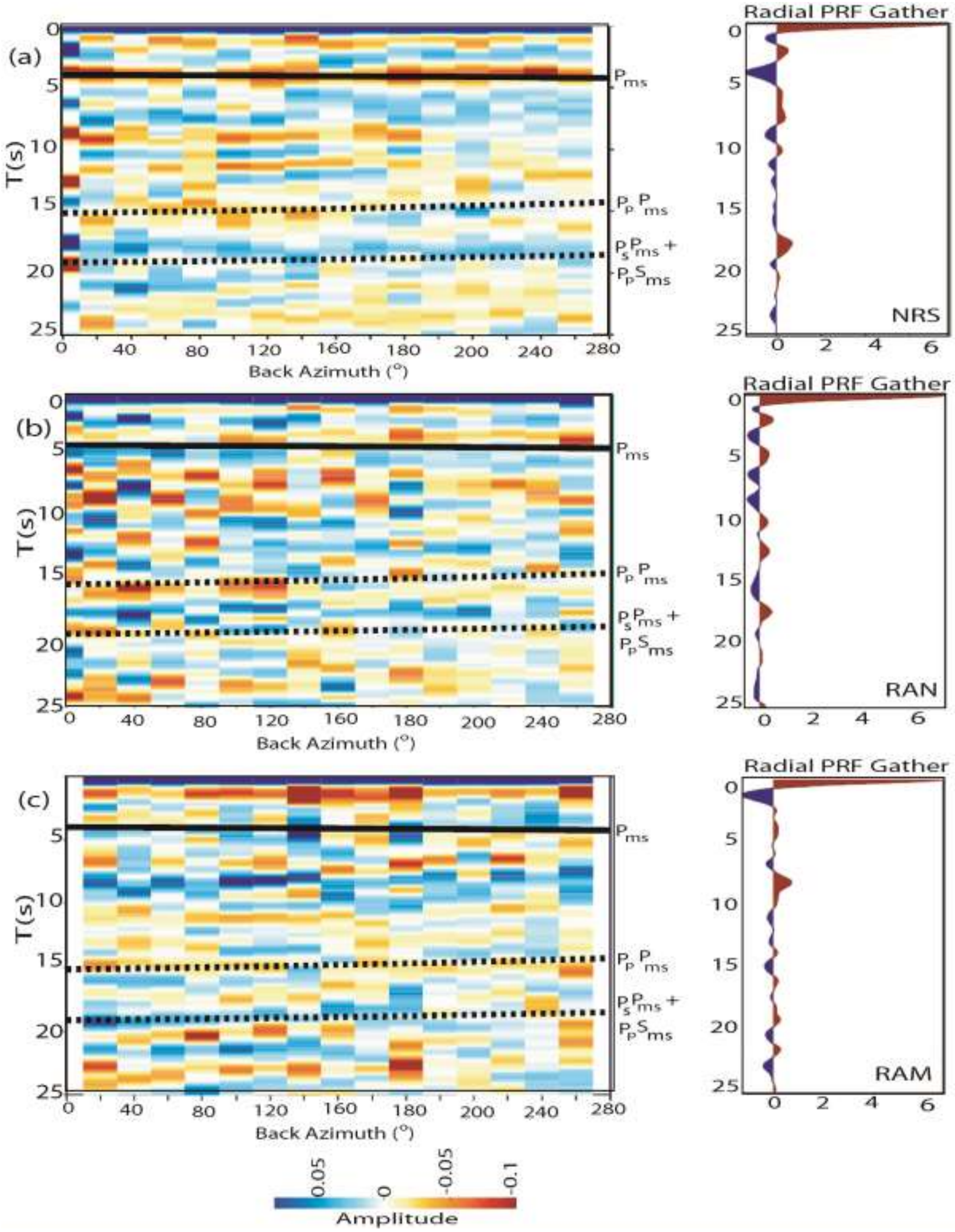

Figure S10: (a) similar to Fig. S7a for the NRS station, (b) similar to Fig. S7a for the RAN station, (c) similar to Fig. S7a for the RAM station.

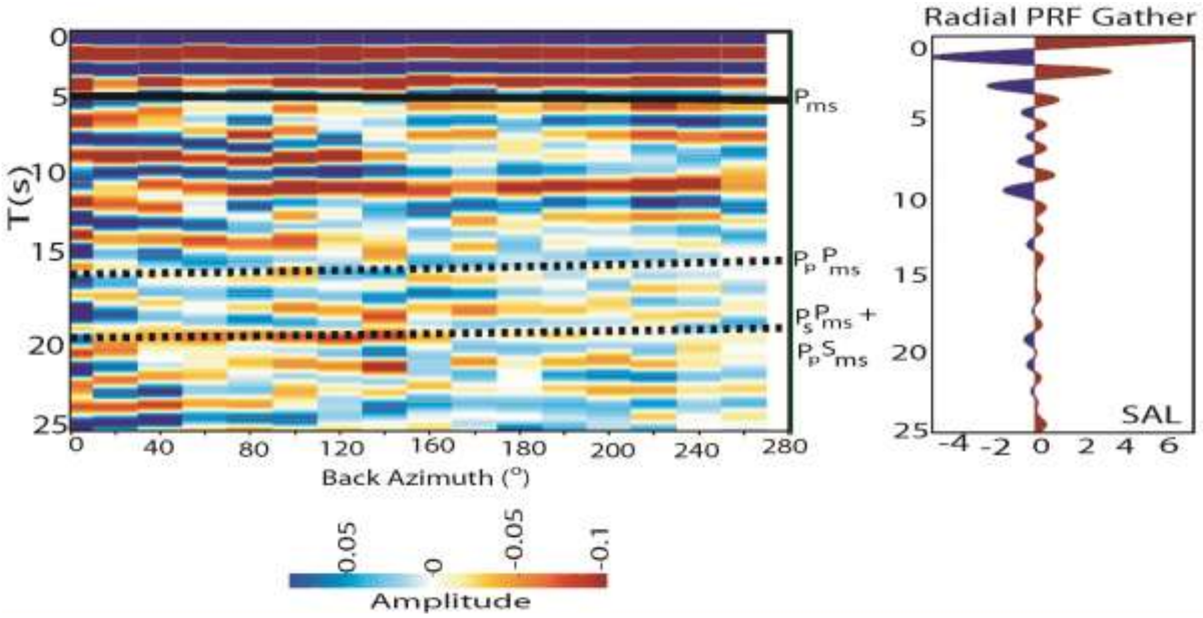

Figure S11: (a) similar to Fig. S7a for the SAL station.

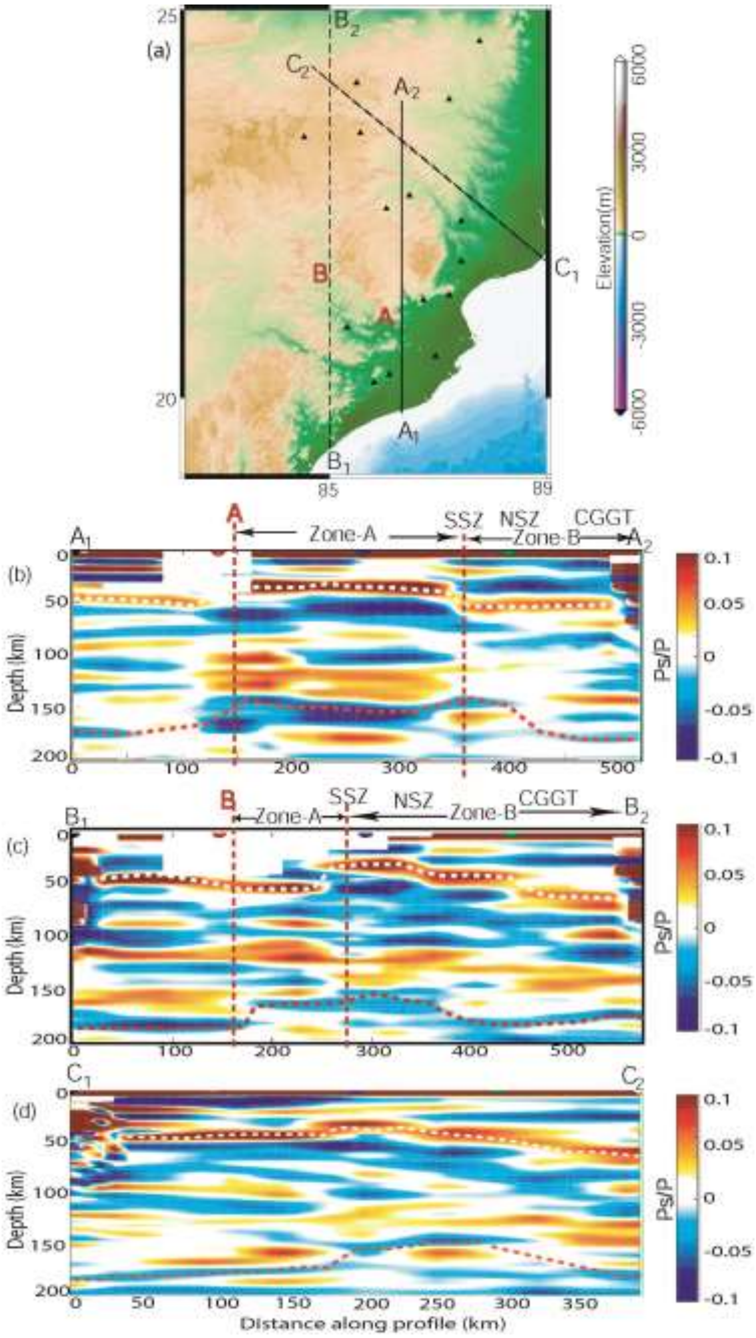

Figure S12: Common Conversion Point (CCP) stacking of radial PRFs (a) along two N-S trending profiles (A_1A_2 and B_1B_2) and one NW-SE trending profile (C_1C_2), (b-d) CCP images along A_1A_2, B_1B_2 and C_1C_2 profiles, respectively, showing a north dipping subduction of Singhbhum craton below the CGGT. Yellow dotted line marks the Moho depth variation while red dotted line shows the variation of lithosphere-asthenosphere boundary across the Eastern Indian Shield (EIS). CTZ shows the region (shown by black dotted lines) with marked crustal and lithospheric thinning that spatially correlates well with the region-A between the point A and the SSZ on the profile A1A2 and the point B and the SSZ on the profile B1B2. While Zone B between the SSZ and the end of the profile A1A2 and B1B2 is characterized by the thickening of the crust and lithosphere suggesting the probable Archean subduction zone of the SOC below the CGGT.
